# Novel Single Nucleotide Polymorphisms (SNPs) and Genetic Features of the Prion Protein Gene (*PRNP*) in Quail (*Coturnix japonica*)

**DOI:** 10.3389/fvets.2022.870735

**Published:** 2022-05-25

**Authors:** Yoonhee Kim, Yong-Chan Kim, Byung-Hoon Jeong

**Affiliations:** ^1^Korea Zoonosis Research Institute, Jeonbuk National University, Iksan, South Korea; ^2^Department of Bioactive Material Sciences and Institute for Molecular Biology and Genetics, Jeonbuk National University, Jeonju, South Korea

**Keywords:** quail, quail (*Coturnix japonica*), prion, PRNP, SNP, polymorphism

## Abstract

Prion diseases are fatal infectious diseases caused by conformational changes of a prion protein (PrP^Sc^) derived from a normal prion protein (PrP^C^). Prion diseases have been reported in several mammalian hosts but not in any birds, including the most popular poultry species, of which chickens showed some resistance to experimental prion infection. To identify the genetic polymorphisms in the quail prion protein gene (*PRNP)*, polymerase chain reaction and DNA sequencing were performed with gene-specific primers in 164 quails. Four *in silico* programs, including PROVEAN, PANTHER, SIFT, and AMYCO, were used to investigate the effect of non-synonymous single nucleotide polymorphisms (SNPs) on quail PrP. Furthermore, to investigate the genetic relationship of avian PrPs, phylogenetic analysis and multiple sequence alignments were performed using MEGA X program. Finally, the secondary and tertiary structures of avian PrPs were analyzed by SWISS-MODEL. We identified 33 novel SNPs in the quail *PRNP* gene, including three non-synonymous SNPs, c.56C>T (T19I), c.60C>T (V21I), and c.61G>A (A22S). Although V21I was predicted to have deleterious effects by SIFT, the substitutions of all three amino acids did not affect the amyloid propensity, 3D structure, or hydrogen bonds of quail PrP. Quail PrP showed a close evolutionary relationship and similar secondary and tertiary structures to chicken PrP compared to duck PrP. To our knowledge, this is the first report on the genetic and structural properties of the quail *PRNP* gene.

## Introduction

Prion diseases are fatal infectious neurodegenerative diseases characterized by spongiform vacuolation and activated glial cells (1). To date, there is no suitable treatment or vaccine to prevent prion diseases. Prion diseases are caused by the β-sheet rich and aggregated form of the prion protein (PrP^Sc^), which originates from the endogenous benign form of the prion protein (PrP^C^) (2). Prion diseases show wide host ranges in several mammals, including Creutzfeldt–Jakob disease (CJD), Gerstmann-Sträussler-Scheinker syndrome (GSS), and fatal familial insomnia (FFI) in humans, bovine spongiform encephalopathy (BSE) in cattle, chronic wasting disease (CWD) in elks and deer, scrapie in sheep and goats, and camel prion disease (CPD) in camels (3–6).

Previous studies have reported that genetic polymorphisms affecting the structure and expression level of PrP are significantly related to susceptibility to prion diseases (7, 8). In humans, non-synonymous single nucleotide polymorphisms (SNPs) at codons 129 and 219 of the prion protein gene (*PRNP*) were associated with susceptibility to CJD (9–11). In addition, the 23 and 12 bp insertion/deletion polymorphisms located in the promoter region were associated with the expression of the bovine *PRNP* gene and were reported to affect vulnerability to BSE (12). Furthermore, genetic polymorphisms at codons 95, 96, and 132 of the cervid *PRNP* gene played an important role in susceptibility to CWD in the cervidae family (13–15). Recent studies have reported that prion disease-resistant specific amino acids of PrPs, including S167 in horses and D159 in dogs, conferred resistance to conversion from PrP^C^ to PrP^Sc^ (16, 17).

In chicken PrP, although the sequence similarity is relatively low with human PrP (approximately 60%), the major domains, including tandem repeat regions and glycosylation sites, are genetically conserved between the two species (18, 19). In addition, the mRNA expression pattern of the *PRNP* gene in the central nervous system is similar in both mammals and birds. Notably, chickens were found to be resistant to experimental BSE infection (20). In addition, chickens have no SNPs in the open reading frame (ORF) of the *PRNP* gene, which may affect the structural properties of PrP (21). In contrast, duck, which show an evolutionarily close relationship with chickens, has additional β-sheet structures in its PrP compared to chicken PrP. Furthermore, duck PrP showed a higher amyloid propensity than chicken PrP, and duck-specific amino acids conferred a high amyloid propensity to chicken PrP (22). Thus, it is necessary to investigate the genetic characteristics related to prion diseases in birds.

In a previous genomic study, microsatellite markers of quail showed significant sequence identities with those of chickens despite 35 million years of their divergence (23). In addition, among the above 20 species of quails around the world, 14 million Japanese quails (*Coturnix japonica*) are bred for eggs and meat in Korea. Although quail is eaten directly, investigation of the genetic properties of the quail *PRNP* gene related to prion diseases has not been performed. Thus, the purpose of this study was to investigate the genetic characteristics of the quail *PRNP* gene in 164 Japanese quails.

## Materials and Methods

### Ethical Statements

A total of 164 Japanese quail samples were obtained from slaughterhouses in the Republic of Korea. All experimental protocols were approved by the Institutional of Animal Care and Use Committee (IACUC) of Jeonbuk National University (JBNU 2021-049). All efforts were made to minimize the number of animals used and their suffering.

### Genomic DNA Extraction

Genomic DNA was extracted from 20 mg peripheral tissues of quails using BioFACT Multi-Bead™ genomic DNA prep kit (BioFACT, Daejeon, Korea) following the manufacturer's instructions.

### Genetic Analysis of the Quail *PRNP* Gene

To amplify quail *PRNP* gene, gene-specific primers were designed and synthesized based on the quail *PRNP* gene registered in GenBank (GeneID: 107323677). The information on gene-specific primers of the quail *PRNP* gene was as follows, forward: 5'-AGGTCTATGCTCGCTGCTCT-3'; and reverse: 5'-AAGGACAAGGGACACCCCAT-3'. These primers were used to amplify the entire ORF of the quail *PRNP* gene. Polymerase chain reaction (PCR) was performed with 2.5 μl of 10 x *Taq* polymerase reaction buffer (25 mM), 5 μl of 5 x band helper, 0.5 μl of dNTPs (each 10 mM), 1 μl of each forward and reverse primers (each 10 μM), 0.12 μl of *Taq* polymerase (5 U/μl) (BioFACT, Daejeon, Korea), 3 μl of template genomic DNA (25-30 ng/μl), and 11.88 μl of nuclease-free water to a total volume of 25 μl. The PCR conditions were as follows: 1 cycle of predenaturation at 98°C for 2 min, 34 cycles of denaturation at 95°C for 20 s, annealing at 60°C for 40 s, elongation at 72°C for 1 min 30 s, and 1 cycle of final elongation at 72°C for 5 min. The experiments were performed using an S-1000 Thermal Cycler (Bio–Rad, Hercules, CA, USA). The PCR product stained with ethidium bromide (EtBr) was loaded to a gel electrophoresis system and then eluted by the Favor prep™ GEL/PCR purification kit (FAVORGEN, PingTung, Taiwan). The purified PCR products were sequenced using BigDye Terminator cycle sequencing kit (ABI, Foster City, CA, USA). The fluorescent PCR protocol consists of an initial 60 s incubation at 96°C, followed by 25 cycles of 96°C for 10 s, 50°C for 5 s, and 60°C for 75 s. Then, PCR products were loaded into an ABI 3730 Capillary Sequencer (ABI, Foster City, CA, USA). The sequencing results were read by the Finch TV program (Geospriza Inc., Seattle, WA, USA), and genotyping was carried out.

### Statistical Analysis

Linkage disequilibrium (LD) and haplotype analyses were performed using Haploview program ver. 4.2 (Broad Institute, Cambridge, MA, USA).

### *In silico* Evaluation of the Effect of Amino Acid Substitution

The effect of amino acid substitution was estimated by four *in silico* programs, including Protein Variation Effect Analyzer (PROVEAN), Protein Analysis Through Evolutionary Relationships (PAHTHER), Sorting Intolerant From Tolerant (SIFT) programs, and combined Amyloid and Composition-based prediction of the prion-like aggregation propensity (AMYCO) (24–27). PROVEAN (http://provean.jcvi.org/seq_submit.php) predicts the effect of amino acid substitution on the biological function of proteins and is scored by calculating clustering BLAST hits of homologs collected from the NCBI database. When the score is < -2.5, it is predicted as “deleterious,” and when it is above −2.5, it is predicted as “neutral.” PANTHER (http://www.pantherdb.org) estimates the likelihood of a non-synonymous SNP to cause a functional impact on the protein. It evaluates amino acid substitution by calculating the time conserved during the evolution of species. Scores based on retention time are as follows: “most likely damaging” if >450 my (~0.2), “possibly damaging” for scores between 200 my and 450 my (~0.4), and “most likely benign” for <200 my. SIFT (https://sift.bii.a-star.edu.sg/) predicts substitution effects based on sequence homology, and the prediction results are described as follows: normalized probabilities ≤ 0.05 are predicted to be damaging, whereas those with normalized probabilities >0.05 are predicted to be tolerated. AMYCO (http://bioinf.uab.es/amycov04/) predicts the effect of amino acid substitution aggregation propensity. It uses the pWALTZ and PAPA algorithms and calculates the results with the PSEP score. An AMYCO score below 0.45 indicates low amyloid propensity while an AMYCO score above 0.74 indicates high amyloid propensity.

### Phylogenetic Analysis

To analyze the genetic relationship, phylogenetic analysis was performed in five species, including chicken, turkey, goose, duck, and quail using Molecular Evolutionary Genetic Analysis X (MEGA X) program (28). Detailed information on the species analyzed in this study is described in Table 1. The evolutionary tree was drawn using neighbor joining method with 2,000 bootstrap tests. The branch length of phylogenetic tree represents the evolutionary distance, which was calculated amino acid substitution by Poisson correction method.

**Table 1:** Detailed information on the avian sequences analyzed in this study.

**Common name**	**Scientific name**	**GenBank ID**
Chicken	*Gallus gallus*	NP_990796.2
Turkey	*Meleagris gallopavo*	XP_003212430.1
Goose	*Anser cygnoides*	XP_013054700.1
Duck	*Anas platyrhynchos domestica*	Jeong et al. [21]
Quail	*Coturnix japonica*	XP_015738493.1

### Multiple Sequence Alignment

To find quail-specific amino acids, multiple sequence alignment was performed in the above-mentioned five avian species using Clustal Omega (29) (https://www.ebi.ac.uk/Tools/msa/clustalo/).

### SWISS-Model

The 3D structure of avian PrPs was analyzed by SWISS-MODEL program (30), with the highest global model quality estimation (GMQE) score selected to predict avian PrPs. The modeling quality was estimated by a qualitative model energy analysis (QMEAN) score. Hydrogen bond analysis was performed to calculate the change in the bond or distance of the hydrogen bond due to amino acid substitution. Hydrogen bonds were predicted if hydrogen was in the 1.2 to 2.76 Å range for a compatible donor atom.

### AlphaFold

To validate the 3D structure of avian PrPs predicted by SWISS-Model, avian PrPs were analyzed by AlphaFold based on machine learning (https://colab.research.google.com/github/deepmind/alphafold/blob/main/notebooks/AlphaFold.ipynb). The intra-domain confidence of modeling was estimated by predicted local distance difference test (pLDDT) score on a scale from 0 to 100.

## Results

### Identification of Novel Genetic Polymorphisms of the Quail *PRNP* Gene

**Figure 1:**
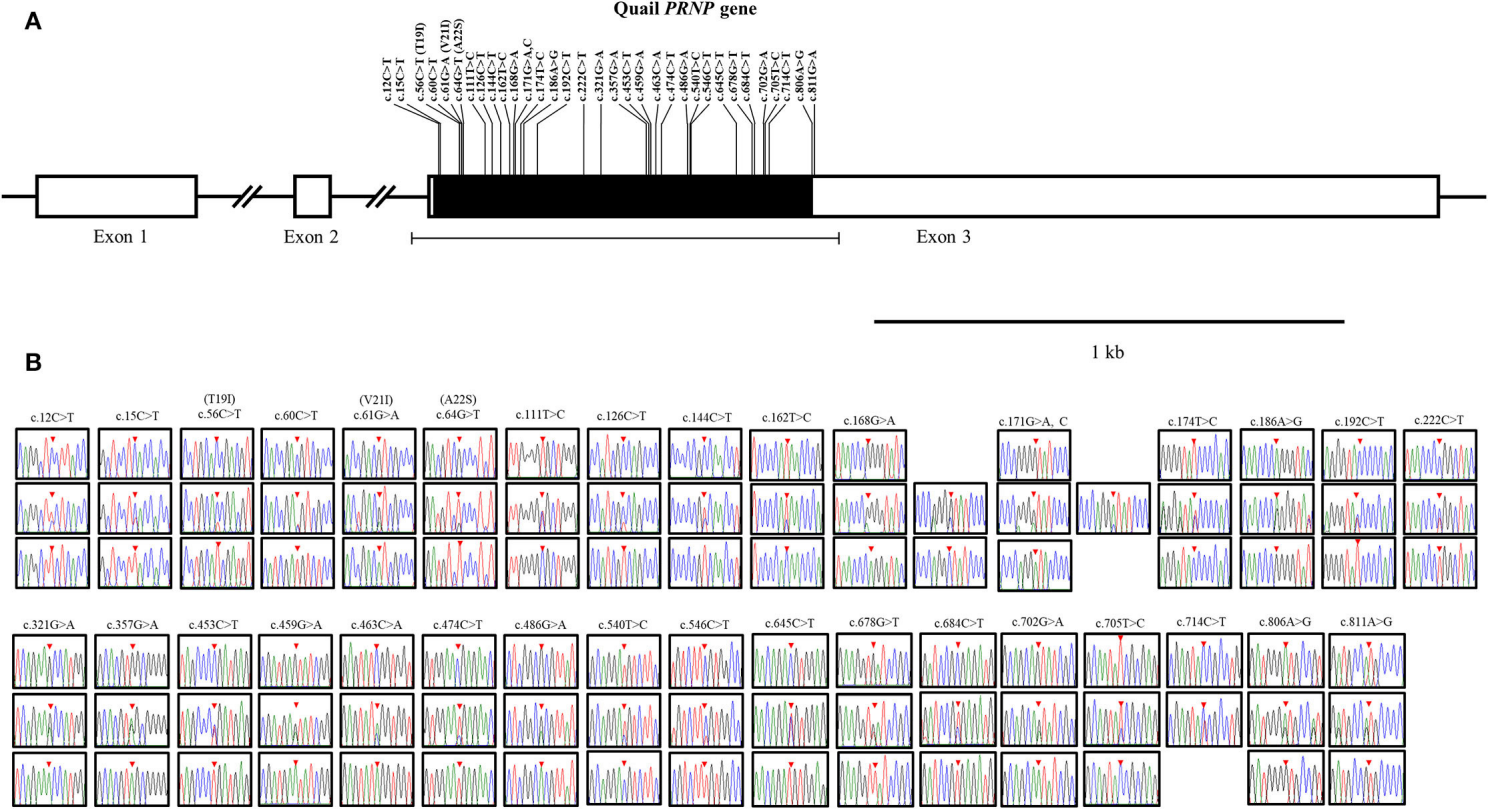
Novel single nucleotide polymorphisms (SNPs) in the quail prion protein gene (*PRNP*) were found in this study. **(A)** Schematic map of the quail *PRNP* gene with SNPs. T19I, V21I, and A22S indicate three nonsynonymous SNPs. **(B)** Electropherograms of the 33 novel SNPs in the quail *PRNP* gene. The colors of the peaks indicate each base of the DNA sequence (green: adenine; red: thymine; blue: cytosine; and black: guanine). The red arrows indicate the location of the SNPs found in this study.

To amplify the quail *PRNP* gene, PCR was performed with gene-specific primers. The quail *PRNP* gene is located on chromosome 22 and consists of three exons. The ORF is located at exon 3. The size of our PCR products (801 bp) was identical to that of the quail *PRNP* gene registered in GenBank (GeneID: 107323677). To identify the genetic polymorphisms of the quail *PRNP* gene, we performed DNA sequencing in 164 quails. We found a total of 33 novel SNPs, including 28 SNPs at c.12C>T, c.15C>T, c.60C>T, c.111T>C, c.126C>T, c.144C>T, c.162T>C, c.168G>A, c.171G>A,C, c.174T>C, c.186A>G, c.192C>T, c.222C>T, c.321G>A, c.357G>A, c.453C>T, c.459G>A, c.463C>A, c.474C>T, c.486G>A, c.540T>C, c.546C>T, c.645C>T, c.678G>T, c.685C>T, c.702G>A, c.705T>C, and c.714C>T, along with three nonsynonymous SNPs at c.56C>T (T19I), c.61G>A (V21I), and c.64G>T (A22S) in the ORF region and two SNPs at c.806A>G and 811G>A in the 3' untranslated region (UTR) (Figure 1A). Electropherograms of the 33 novel SNPs are shown in Figure 1B. Notably, insertion/deletion polymorphisms were not found in the quail *PRNP* gene. Detailed information on the genotype and allele frequencies of the 33 SNPs of the quail *PRNP* gene is described in Table 2. In addition, we performed LD analysis to evaluate genetic linkage among 33 SNPs of the quail *PRNP* gene using the *r*^2^ value. Detailed LD values are described in Table 3. Furthermore, we performed haplotype analysis on the 33 SNPs of the quail *PRNP* gene. Briefly, seven major haplotypes were found. The haplotype present in the highest proportion was CCCCGGTCCTGGTACCGGCGCCGTCCGCGTCAG (62.5%), followed by CCCCAGCCCTAATGTCAACACCACCCTCATCAG (5.2%) and CCTTGTTCCCAACACCGATGCCGTCTGCGCCAA (2.1%) (Table 4).

**Table 2:** Genotype and allele frequencies of the single nucleotide polymorphisms in the quail prion protein gene (*PRNP*).

**Polymorphism**	**Genotype frequency;** ***n*** **(%)**			**Allele frequency;** ***n*** **(%)**	
	**M/M**	**M/m**	**m/m**	**M**	**m**
c.12C>T	152 (92.68)	3 (1.83)	9 (5.49)	307 (93.60)	21 (6.40)
c.15C>T	160 (97.56)	2 (1.22)	2 (1.22)	322 (98.17)	6 (1.83)
c.56C>T (T19I)	137 (83.54)	15 (9.15)	12 (7.31)	289 (88.11)	39 (11.89)
c.60C>T	141 (85.97)	15 (9.15)	8 (4.88)	297 (90.55)	31 (9.45)
c.61G>A (V21I)	138 (84.15)	16 (9.76)	10 (6.09)	292 (89.02)	36 (10.98)
c.64G>T (A22S)	137 (83.54)	17 (10.37)	10 (6.09)	291 (88.72)	37 (11.28)
c.111T>C	120 (73.17)	27 (16.46)	17 (10.37)	267 (81.40)	61 (18.60)
c.126C>T	157 (95.73)	2 (1.22)	5 (3.05)	316 (96.34)	12 (3.66)
c.144C>T	150 (91.46)	7 (4.27)	7 (4.27)	307 (93.60)	21 (6.40)
c.162T>C	142 (86.58)	15 (9.15)	7 (4.27)	299 (91.16)	29 (8.84)
c.168G>A	103 (62.81)	30 (18.29)	31 (18.90)	236 (71.95)	92 (28.05)
c.171G>A; C	109 (66.46)	28 (17.07)	27 (16.47)	298 (90.85)	30 (9.15)
c.174T>C	146 (89.02)	9 (5.49)	9 (5.49)	301 (91.77)	28 (8.23)
c.186A>G	151 (92.07)	3 (1.83)	10 (6.10)	305 (92.99)	23 (7.01)
c.192C>T	133 (81.10)	21 (12.80)	10 (6.10)	287 (87.50)	41 (12.5)
c.222C>T	158 (96.34)	5 (3.05)	1 (0.61)	321 (97.87)	7 (2.13)
c.321G>A	140 (85.37)	14 (8.53)	10 (6.10)	294 (89.63)	34 (10.37)
c.357G>A	98 (59.76)	33 (20.12)	33 (20.12)	229 (69.82)	99 (30.18)
c.453C>T	140 (85.37)	17 (10.36	7 (4.27)	297 (90.55)	31 (9.45)
c.459G>A	133 (81.10)	21 (12.80)	10 (6.10)	287 (87.50)	41 (12.50)
c.463C>A	161 (98.17)	2 (1.22)	1 (0.61)	324 (98.78)	4 (1.22)
c.474C>T	161 (98.17)	2 (1.22)	1 (0.61)	324 (98.78)	4 (1.22)
c.486G>A	133 (81.10)	21 (12.80)	10 (6.10)	287 (87.50)	41 (12.50)
c.540T>C	131 (79.88)	23 (14.02)	10 (6.10)	285 (86.89)	43 (13.11)
c.546C>T	157 (95.73)	6 (3.66)	1 (0.61)	320 (97.56)	8 (2.44)
c.645C>T	142 (86.58)	16 (9.76)	6 (3.66)	300 (91.46)	28 (8.54)
c.678G>T	136 (82.93)	18 (10.97)	10 (6.10)	290 (88.42)	38 (11.58)
c.684C>T	162 (98.78)	1 (0.61)	1 (0.61)	325 (99.09)	3 (0.91)
c.702G>A	132 (80.49)	19 (11.59)	13 (7.92)	283 (86.28)	45 (13.72)
c.705T>C	144 (87.81)	13 (7.92)	7 (4.27)	301 (91.77)	27 (8.23)
c.714C>T	152 (92.68)	12 (7.32)	0 (0)	316 (96.34)	12 (3.66)
c.806A>G	158 (96.93)	2 (1.23)	3 (1.84)	318 (98.15)	6 (1.85)
c.811G>A	143 (87.73)	17 (10.43)	3 (1.84)	303 (92.95)	23 (7.05)

*MM, major homozygote; Mm: heterozygote; mm, minor homozygote; M, major allele; and m: minor allele*.

**Table 3:** Linkage disequilibrium (LD) score of the single nucleotide polymorphisms in the quail prion protein gene (*PRNP*).

	**1**	**2**	**3**	**4**	**5**	**6**	**7**	**8**	**9**	**10**	**11**	**12**	**13**	**14**	**15**	**16**	**17**	**18**	**19**	**20**	**21**	**22**	**23**	**24**	**25**	**26**	**27**	**28**	**29**	**30**	**31**	**32**	**33**
**1**	-																																
**2**	0.023	-																															
**3**	0.002	0.051	-																														
**4**	0.007	0.069	**0.663**	-																													
**5**	0.008	0.002	0.009	0.013	-																												
**6**	0.009	0.055	**0.776**	**0.705**	0.016	-																											
**7**	0.147	0.01	0.009	0.024	**0.502**	0.011	-																										
**8**	0.0	0.21	0.107	0.106	0.005	0.151	0.001	-																									
**9**	0.24	0.023	0.009	0.007	0.0	0.009	0.2	0.003	-																								
**10**	0.007	0.0	**0.609**	**0.495**	0.012	**0.647**	0.022	0.0	0.007	-																							
**11**	0.153	0.09	0.205	0.134	0.29	0.208	**0.558**	0.001	0.108	0.249	-																						
**12**	0.155	0.013	0.247	0.164	0.173	0.249	**0.386**	0.001	0.107	0.291	**0.826**	-																					
**13**	0.138	0.016	0.126	0.117	0.008	0.089	0.072	0.03	0.177	0.016	0.183	0.216	-																				
**14**	0.005	0.001	0.01	0.008	**0.612**	0.01	0.33	0.003	0.005	0.007	0.193	0.153	0.007	-																			
**15**	0.0	0.003	0.019	0.015	**0.705**	0.005	**0.514**	0.0	0.001	0.014	**0.315**	0.245	0.0	**0.528**	-																		
**16**	0.001	**0.37**	0.111	0.095	0.003	0.118	0.005	**0.416**	0.001	0.0	0.006	0.001	0.001	0.002	0.003	-																	
**17**	0.008	0.002	0.008	0.012	**0.877**	0.007	**0.469**	0.004	0.0	0.011	0.297	0.174	0.006	**0.652**	**0.653**	0.003	-																
**18**	0.137	0.043	0.264	0.174	0.261	0.27	**0.503**	0.035	0.114	0.201	**0.846**	**0.717**	0.144	0.174	**0.306**	0.028	0.244	-															
**19**	0.005	0.0	**0.611**	**0.506**	0.013	**0.649**	0.024	0.002	0.007	**0.859**	0.268	**0.313**	0.117	0.008	0.006	0.003	0.012	0.241	-														
**20**	0.01	0.003	0.008	0.015	**0.863**	0.007	0.55	0.005	0.001	0.01	**0.315**	0.183	0.006	**0.528**	**0.788**	0.003	**0.81**	**0.306**	0.014	-													
**21**	0.18	0.0	0.002	0.001	0.002	0.012	0.054	0.012	0.001	0.001	0.032	0.037	0.001	0.001	0.0	0.0	0.001	0.029	0.001	0.002	-												
**22**	0.18	0.0	0.002	0.001	0.002	0.012	0.054	0.012	0.001	0.001	0.032	0.037	0.001	0.001	0.0	0.0	0.001	0.029	0.001	0.002	**1.0**	-											
**23**	0.01	0.003	0.008	0.015	**0.863**	0.007	0.55	0.005	0.001	0.01	**0.315**	0.183	0.006	**0.528**	**0.788**	0.003	**0.81**	**0.306**	0.014	**1.0**	0.002	0.002	-										
**24**	0.005	0.003	0.01	0.016	**0.715**	0.004	**0.585**	0.006	0.001	0.005	**0.361**	0.224	0.009	**0.451**	**0.697**	0.003	**0.715**	**0.324**	0.008	**0.843**	0.082	0.082	**0.843**	-									
**25**	0.04	0.0	0.003	0.003	0.003	0.003	0.011	0.002	0.0	0.002	0.004	0.006	0.002	0.0	0.0	0.001	0.002	0.008	0.003	0.004	0.272	0.272	0.004	0.025	-								
**26**	0.007	0.0	**0.582**	**0.463**	0.012	**0.619**	0.021	0.003	0.006	**0.888**	0.239	0.28	0.138	0.07	0.008	0.005	0.011	0.216	**0.894**	0.008	0.001	0.001	0.008	0.004	0.002	-							
**27**	0.009	0.002	0.008	0.014	**0.826**	0.017	**0.536**	0.005	0.0	0.013	**0.31**	0.173	0.011	**0.52**	**0.708**	0.003	**0.768**	0.279	0.009	**0.863**	0.002	0.002	**0.863**	**0.716**	0.003	0.012	-						
**28**	0.135	0.0	0.001	0.001	0.001	0.001	0.04	0.019	0.001	0.001	0.024	0.028	0.001	0.001	0.001	0.0	0.001	0.021	0.001	0.001	**0.748**	**0.748**	0.001	0.061	**0.639**	0.001	0.001	-					
**29**	0.003	0.003	0.006	0.006	**0.775**	0.002	**0.514**	0.006	0.001	0.002	**0.308**	0.189	0.001	**0.474**	**0.751**	0.003	**0.727**	0.296	0.004	**0.898**	0.0	0.0	**0.898**	0.8	0.0	0.002	**0.774**	0.0	-				
**30**	0.003	0.0	**0.556**	**0.485**	0.011	**0.647**	0.002	0.0	0.006	**0.851**	0.23	0.269	0.089	0.007	0.013	0.0	0.01	0.207	**0.859**	0.006	0.001	0.001	0.006	0.002	0.002	**0.811**	0.011	0.001	0.001	-			
**31**	0.0	0.005	0.0	0.002	0.025	0.0	0.065	0.002	0.002	0.003	0.029	0.013	0.003	0.0	0.035	0.003	0.004	0.024	0.002	0.058	0.0	0.0	0.058	0.053	0.001	0.004	0.041	0.0	0.052	0.005	-		
**32**	0.002	**0.321**	0.032	0.045	0.003	0.035	0.0	0.152	0.04	0.001	0.0	0.0	0.001	0.002	0.04	0.271	0.003	0.011	0.002	0.004	0.0	0.0	0.004	0.004	0.001	0.001	0.003	0.0	0.004	0.001	0.003	-	
**33**	0.003	0.0	**0.451**	**0.359**	0.009	**0.479**	0.013	0.0	0.005	**0.704**	0.196	0.226	0.112	0.006	0.011	0.0	0.009	0.177	**0.722**	0.006	0.003	0.003	0.006	0.002	0.002	**0.732**	0.01	0.001	0.001	**0.687**	0.001	0.0	-

*1, c.12C>T; 2, c.15C>T; 3, c.56C>T (T19I); 4, c.60C>T; 5, c.61G>A (V21I); 6, c.64G>T (A22S); 7, c.111T>C; 8, c.126C>T; 9, c.144C>T; 10, c.162T>C; 11, c.168G>A; 12, c.171G>A; 13, c.174T>C; 14, c.186A>G; 15, c.192C>T; 16, c.222C>T; 17, c.321G>A; 18, c.357G>A; 19, c.453C>T; 20, c.459G>A; 21, c.463C>A; 22, c.474C>T; 23, c.486G>A; 24, c.540T>C; 25: c.546C>T; 26, c.645C>T; 27, c.678G>T; 28: c.684C>T; 29, c.702G>A; 30, c.705T>C; 31, c714C>T; 32, c.806A>G; and 33, c.811G>A. Bold font means strong LD (r^2^ > 0.3)*.

**Table 4:** Haplotype frequencies of the quail prion protein gene (PRNP).

	**1**	**2**	**3**	**4**	**5**	**6**	**7**	**8**	**9**	**10**	**11**	**12**	**13**	**14**	**15**	**16**	**17**	**18**	**19**	**20**	**21**	**22**	**23**	**24**	**25**	**26**	**27**	**28**	**29**	**30**	**31**	**32**	**33**	**Frequency (n = 328)**
HT1	C	C	C	C	G	G	T	C	C	T	G	G	T	A	C	C	G	G	C	G	C	C	G	T	C	C	G	C	G	T	C	A	G	205 (0.625)
HT2	C	C	C	C	A	G	C	C	C	T	A	A	T	G	T	C	A	A	C	A	C	C	A	C	C	C	T	C	A	T	C	A	G	17 (0.052)
HT3	C	C	T	T	G	T	T	C	C	C	A	A	C	A	C	C	G	A	T	G	C	C	G	T	C	T	G	C	G	C	C	A	A	7 (0.021)
HT4	C	C	T	T	G	T	T	C	C	C	A	A	T	A	C	C	G	A	T	G	C	C	G	T	C	T	G	C	G	C	C	A	A	6 (0.019)
HT5	T	C	C	C	G	G	C	C	T	T	A	A	C	A	C	C	G	A	C	G	C	C	G	T	C	C	G	C	G	T	C	A	G	6 (0.018)
HT6	C	C	C	C	G	G	T	C	C	T	G	G	T	A	C	C	G	G	C	G	C	C	G	T	T	C	G	C	G	T	C	A	G	5 (0.015)
HT7	C	T	T	T	G	T	T	T	C	T	G	G	T	A	C	T	G	A	C	G	C	C	G	T	C	C	G	C	G	T	C	G	G	4 (0.012)
Others	-	-	-	-	-	-	-	-	-	-	-	-	-	-	-	-	-	-	-	-	-	-	-	-	-	-	-	-	-	-	-	-	-	78 (0.238)

*1, c.12C>T; 2, c.15C>T; 3, c.56C>T (T19I); 4, c.60C>T; 5, c.61G>A (V21I); 6, c.64G>T (A22S); 7, c.111T>C; 8, c.126C>T; 9, c.144C>T; 10, c.162T>C; 11, c.168G>A; 12, c.171G>A; 13, c.174T>C; 14, c.186A>C; 15, c.192C>T; 16, c.222C>T; 17, c.321G>A; 18, c.357G>A; 19, c.453C>T; 20, c.459G>A; 21, c.463C>T; 22, c.474C>T; 23, c.486G>A; 24, c.540T>C; 25, c.546C>T; 26, c.645C>T; 27, c.678G>T; 28, c.684C>T; 29, c.702G>A; 30, c.705T>C; 31, c714C>T; 32, c.806A>G; and 33, c.811G>A*.

### *In silico* Evaluation of the Effect of Non-synonymous SNPs on Quail PrP

**Figure 2:**
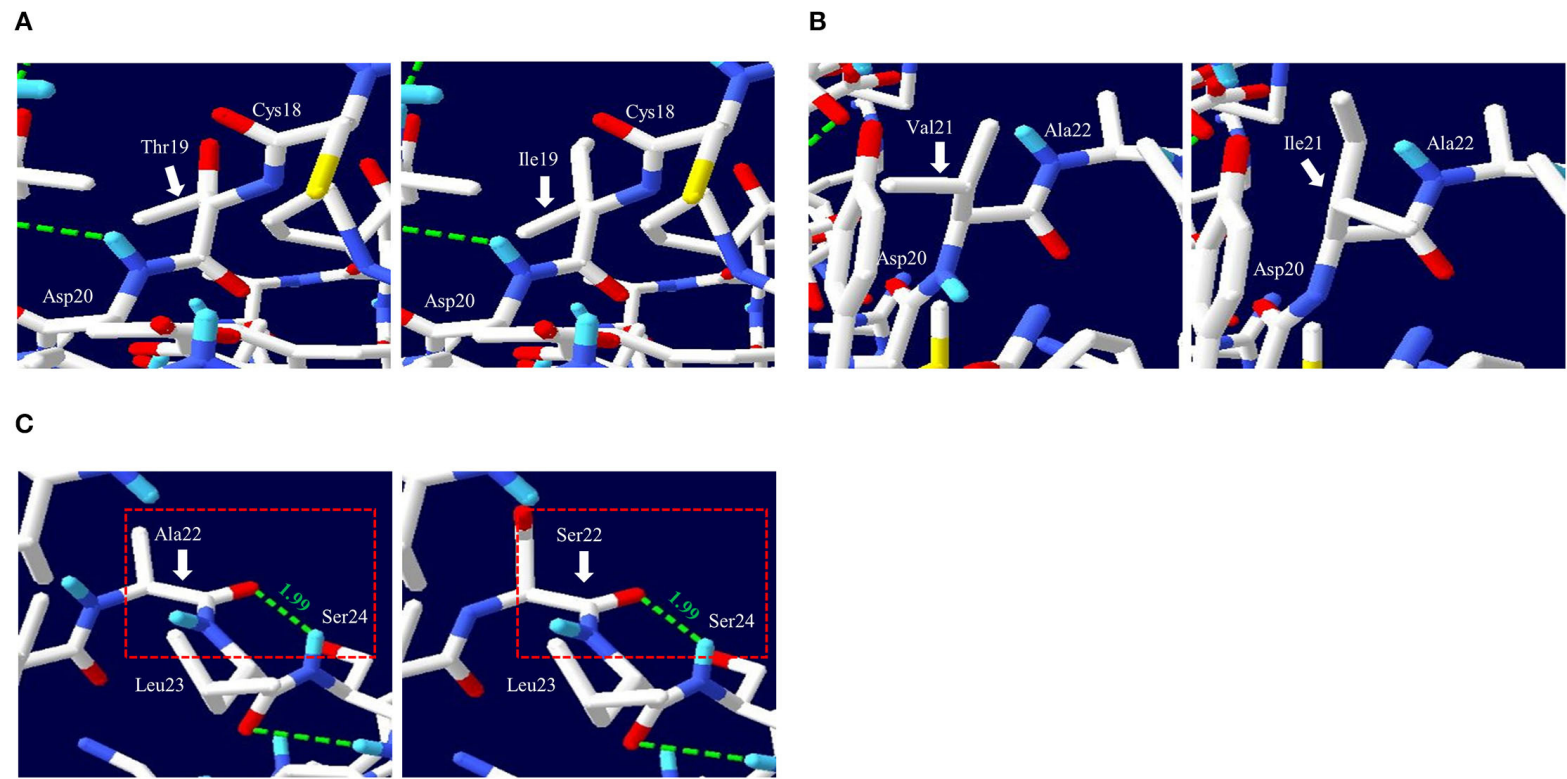
Prediction of tertiary structure and hydrogen bonds of quail prion protein (PrP). **(A)** Comparison of the 3D structure and hydrogen bond of quail PrP according to the T19I alleles. The left panel indicates the 3D structure of quail PrP with allele T19. The right panel indicates the 3D structure of quail PrP with allele I19. **(B)** Comparison of the 3D structure and hydrogen bond of quail PrP according to the V21I alleles. The left panel indicates the 3D structure of quail PrP with allele V21. The right panel indicates the 3D structure of quail PrP with allele I21. **(C)** Comparison of the 3D structure and hydrogen bond of quail PrP according to the A22S alleles. The left panel indicates the 3D structure of quail PrP with allele A22. The right panel indicates the 3D structure of quail PrP with allele S22. The green dotted lines indicate hydrogen bonds. The green numbers indicate the distance of the hydrogen bonds.

To analyze the functional and structural effects of nonsynonymous SNPs on quail PrP, PROVEAN, PANTEHR, and SIFT programs were used. T19I and A22S were predicted to have benign effects on quail PrP by all three programs. In addition, V21I was predicted by PROVEAN and PANTHER to have a neutral effect on quail PrP. However, V21I was predicted to have a deleterious effect on quail PrP by SIFT (Table 5). To analyze the effect of nonsynonymous SNPs on the amyloid propensity of quail PrP, the AMYCO program was used. Notably, T19I, V21I, and A22S were predicted to have low amyloid propensity with a score of “0” (Table 5). T19, I19, V21, and I21 alleles were predicted to have no hydrogen bond. A22 and S22 alleles were predicted to have one hydrogen bond with S24 (1.99 Å) (Figure 2).

**Table 5:** *In silico* evaluation on effect of nonsynonymous single nucleotide polymorphisms in the quail prion protein gene (*PRNP*).

**Polymorphism**	**Method**	**Score**	**Prediction**
c.56C>T(T19I)	PROVEAN PANTHER SIFT AMYCO	−1.266 0.02 0.285 0.0	Neutral Probably benign Tolerated Neutral
c.61G>A(V21I)	PROVEAN PANTHER SIFT AMYCO	−0.453 0.02 0.020 0.0	Neutral Probably benign Damaging Neutral
c.64G>T(A22S)	PROVEAN PANTHER SIFT AMYCO	0.473 0.02 0.480 0.0	Neutral Probably benign Tolerated Neutral

### Analysis of Evolutionary Relationships and Multiple Sequence Alignment of Avian PrPs

**Figure 3:**
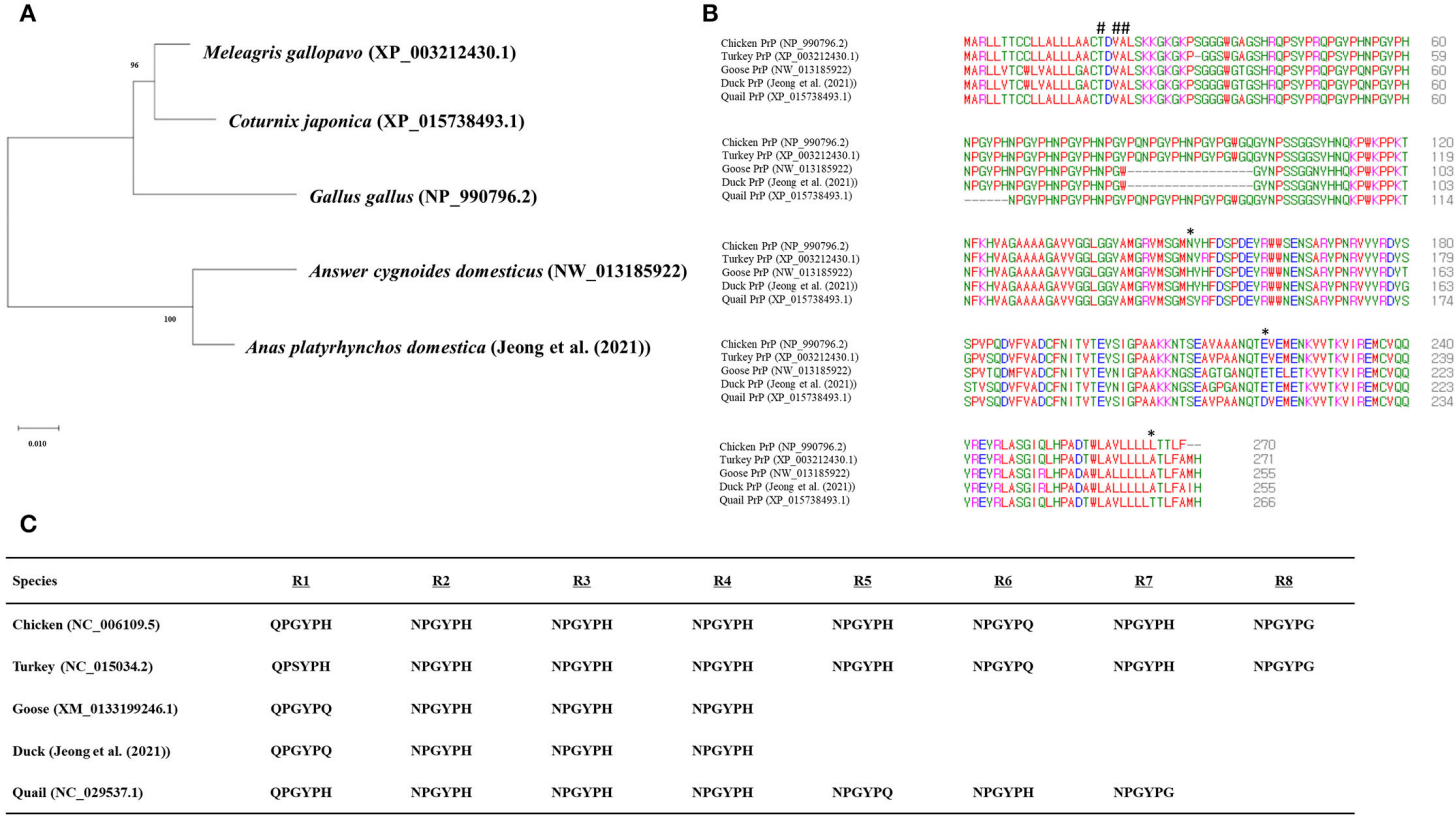
Comparison of the amino acid sequences among five avian PrPs. **(A)** Phylogenetic analysis of the PrPs in the five avian species. **(B)** Multiple sequence alignment of the amino acid sequences of the five avian PrPs. Colors indicate the chemical properties of amino acids, namely, blue: acidic; red: small and hydrophobic magenta: basic; and green: hydroxyl, sulfhydryl, amine, and glycine. Asterisks indicate quail-specific amino acids. Sharps indicate T19I, V21I, and A22S. **(C)** Comparison of tandem repeat sequences in the five avian species. R1 - R8 indicate hexapeptide repeat regions of avian PrPs.

To analyze the evolutionary relationship of quail PrP among avian species, phylogenetic analysis was performed in five avian species of chicken, turkey, goose, duck, and quail using MEGA X program. Notably, quail PrP showed the closest genetic distance to turkey PrP, followed by chicken PrP (Figure 3A). Multiple sequence alignment showed that length of the amino acid sequences of duck PrP was the longest (136 aa), while the length of the amino acid sequence of quail PrP (115 aa) was identical to those of turkey and goose. In addition, we found three quail-specific amino acids, namely S144, D214, and T259 (Figure 3B).

### Comparison of Tandem Repeats of Avian PrPs

To identify the difference in tandem repeats in amino acid sequences among the five avian species, we aligned their tandem repeat regional sequences (Figure 3C). The result showed that both chicken and turkey PrPs had eight tandem repeats, followed by seven in quail and four each in geese and duck.

### Comparison of the Secondary and Tertiary Structures of the Avian PrPs

**Figure 4:**
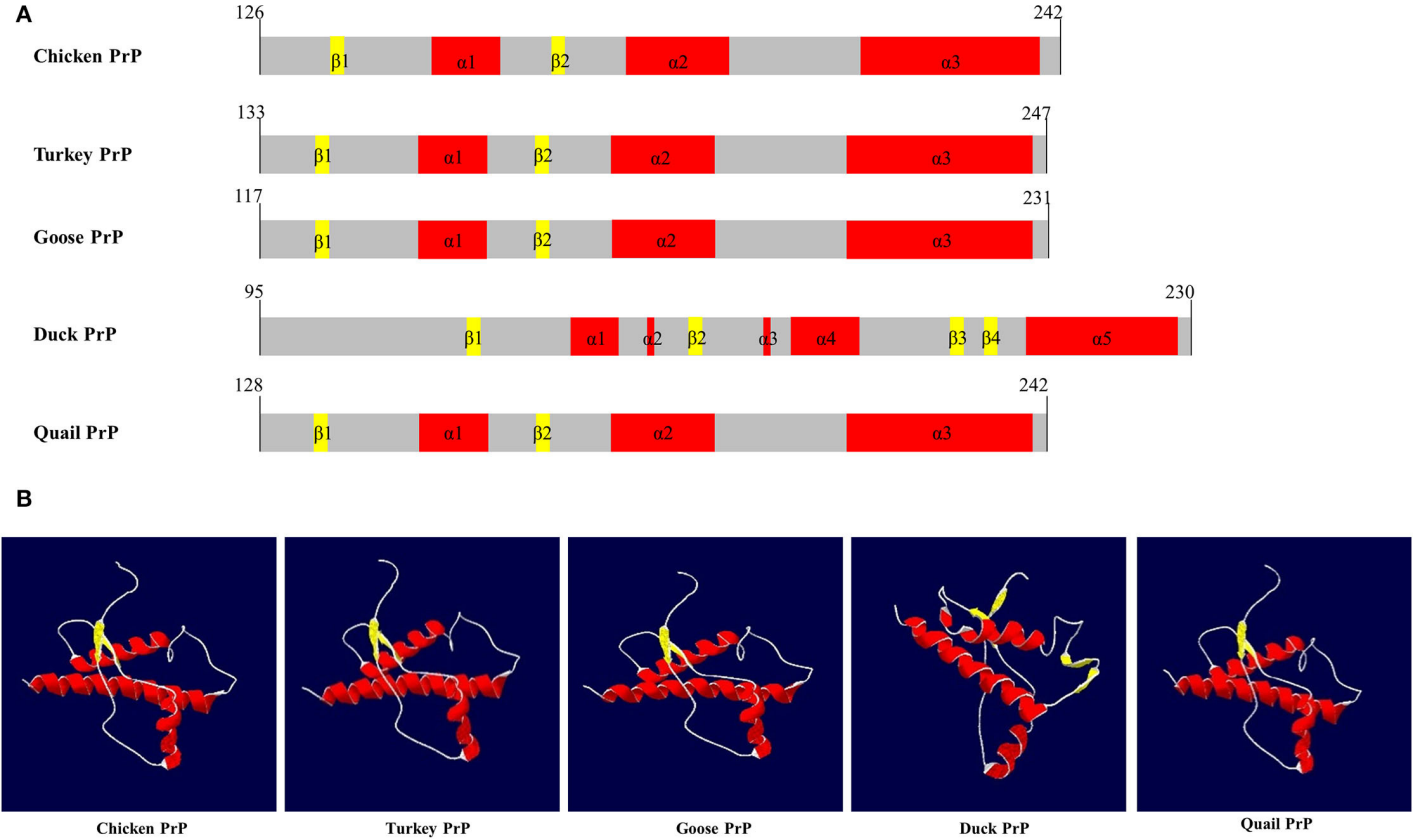
The secondary and tertiary structures of avian PrPs. **(A)** The secondary structure of the avian PrPs. **(B)** The tertiary structure of the avian PrPs. The colors indicate α-helices (red), β-sheets (yellow), and coils (white).

SWISS-MODEL was used to analyze the secondary structure of avain PrPs (Figure 4, Table 6). In chicken, turkey, and goose PrPs, three α-helix and two β-sheet structures were observed, followed by five α-helix and four β-sheet structures in duck, and three α-helix and two β-sheet structures in quail. The proportion of the β-sheet structure of quail PrP was lower than that of duck (quail: 5.21%; duck: 8.82%). As shown in the secondary structure of avian PrPs, all avian PrPs showed similar tertiary structures (Figure 4B, Table 6). Since SWISS-MODEL has been predicted with a relatively low confidence (Table 6), we validated the 3D structure of avian PrPs using AlphaFold (Supplementary Figure 1). Notably, quail PrP showed a similar 3D structure with chicken PrP.

**Table 6:** Detailed information on the secondary and tertiary structures of avian prion proteins (PrPs).

**Species**	**Template**	**QMEAN**	**GMQE**	**Range**	**Information of** **α-helices**			**Information of** **β-sheets**	
					**Location**	**Overall ratio**	**Location**	**Overall ratio**
Chicken	1u3m	-	-	117 (126–242)	151–161, 179–194, 213–240	47.00	136–138, 168–170	5.12
Turkey	1u3m.1	−3.85	0.31	115 (133–247)	156–166, 184–199, 218-245	47.82	141–143, 173–175	5.21
Goose	1u3m.1.A	−4.10	0.38	115 (117–231)	140–150, 168–183, 202–229	47.82	125–127, 157–159	5.21
Duck	2lft.1.A	−3.8	0.37	136 (95–230)	140–147, 151, 168, 172–182, 206–228	32.67	125–127, 157–159, 195–197, 200–202	8.82
Quail	1u3m.1.A	−3.89	0.34	115 (128–242)	151–161, 179–194, 213–240	47.82	136–138, 168–170	5.21

QMEAN, qualitative model energy analysis; GMQE, global model quality estimation.

### Amyloid Propensity of Amino Acid Substitutions

To analyze the amyloid propensity of the substitutions of three amino acids on avian PrPs, we evaluated amyloid propensity according to the allele using AMYCO (Table 7). Among the five avian species, duck PrP was predicted to have the highest amyloid propensity (44) compared to all other four avian PrPs. Notably, the amyloid propensity of the avian PrPs was not changed by any substitution of the three amino acids.

**Table 7:** AMYCO analysis according to alleles of avian PrPs.

	**Chicken**	**Turkey**	**Goose**	**Duck**	**Quail**
Wild type	0.00	0.00	0.00	0.00	0.44
T19I	0.00	0.00	0.00	0.00	0.44
V21I	0.00	0.00	0.00	0.00	0.44
A22S	0.00	0.00	0.00	0.00	0.44

## Discussion

Prion protein is a GPI-anchor protein. Notably, a V21I is located on the signal peptide of PrP, which plays a pivotal role in the trafficking of PrP (31, 32). Previous studies have reported that a block of trafficking of PrP via mutagenesis of PrP's signal peptide affects the resistance to the conversion of PrP^C^ to PrP^Sc^ (33, 34). Since we predicted the deleterious effect of V21I on quail PrP (Table 5), further *in vitro* validation of its impact on the cellular localization of quail PrP using a reporter system is highly desirable in the future.

In previous studies, insertion/deletion polymorphisms of the *PRNP* gene have been related to susceptibility to several types of prion diseases (35, 36). In humans, insertion polymorphisms of octapeptide repeats of the *PRNP* gene are more frequently observed in patients with human genetic prion disease. Furthermore, 23- and 12-bp insertion/deletion polymorphisms regulate the expression level of the bovine *PRNP* gene and are associated with susceptibility to classical BSE. Furthermore, we found strong LD among six SNPs (Table 3), indicating a clear LD block in the ORF of the *PRNP* gene.

## Conclusion

In this study, we found 33 novel SNPs in the quail *PRNP* gene. We predicted V21I to have a deleterious effect on quail PrP. In addition, quail PrP showed a close evolutionary relationship and similar secondary and tertiary structures to chicken PrP. To our knowledge, this was the first report on the genetic and structural properties of the quail *PRNP* gene.

## Data Availability Statement

The datasets presented in this study can be found in online repositories. The names of the repository/repositories and accession number(s) can be found in the article/Supplementary Material.

## Ethics Statement

The animal study was reviewed and approved by Institutional Animal Care and Use Committee of Jeonbuk National University.

## Author Contributions

YK, Y-CK, and B-HJ conceived and designed the experiment, analyzed the data, and wrote the paper. YK and Y-CK performed the experiments. All authors read and approved the final manuscript.

## Funding

YK supported by the BK21 Plus Program in the Department of Bioactive Material Sciences. This research was supported by the Basic Science Research Program through the National Research Foundation (NRF) of Korea funded by the Ministry of Education (2017R1A6A1A03015876 and 2021R1A6A3A010864), the National Research Foundation of Korea (NRF) grant funded by the Korean government (MSIT) (2021R1A2C1013213), and a National Research Foundation of Korea (NRF) grant funded by the Korean Government (NRF-2019-Fostering Core Leaders of the Future Basic Science Program/Global Ph.D. Fellowship Program).

## Conflict of Interest

The authors declare that the research was conducted in the absence of any commercial or financial relationships that could be construed as a potential conflict of interest.

## Publisher's Note

All claims expressed in this article are solely those of the authors and do not necessarily represent those of their affiliated organizations, or those of the publisher, the editors and the reviewers. Any product that may be evaluated in this article, or claim that may be made by its manufacturer, is not guaranteed or endorsed by the publisher.

## Supplementary Material

Supplementary Figure 1 **(A)** The tertiary structure of the chicken prion protein (PrP). **(B)** The tertiary structure of turkey PrP. **(C)** The tertiary structure of the goose PrP. **(D)** The tertiary structure of duck PrP. **(E)** The tertiary structure of the quail PrP. pLDDT: predicted local distance difference test.
