# Supplementary figures and images for: Novel Single Nucleotide Polymorphisms (SNPs) and Genetic Features of the Prion Protein Gene (PRNP) in Quail (Coturnix japonica)
Source: Front Vet Sci. 2022 May 25;9:870735. doi: 10.3389/fvets.2022.870735 (PMC9174905; doi:10.3389/fvets.2022.870735)

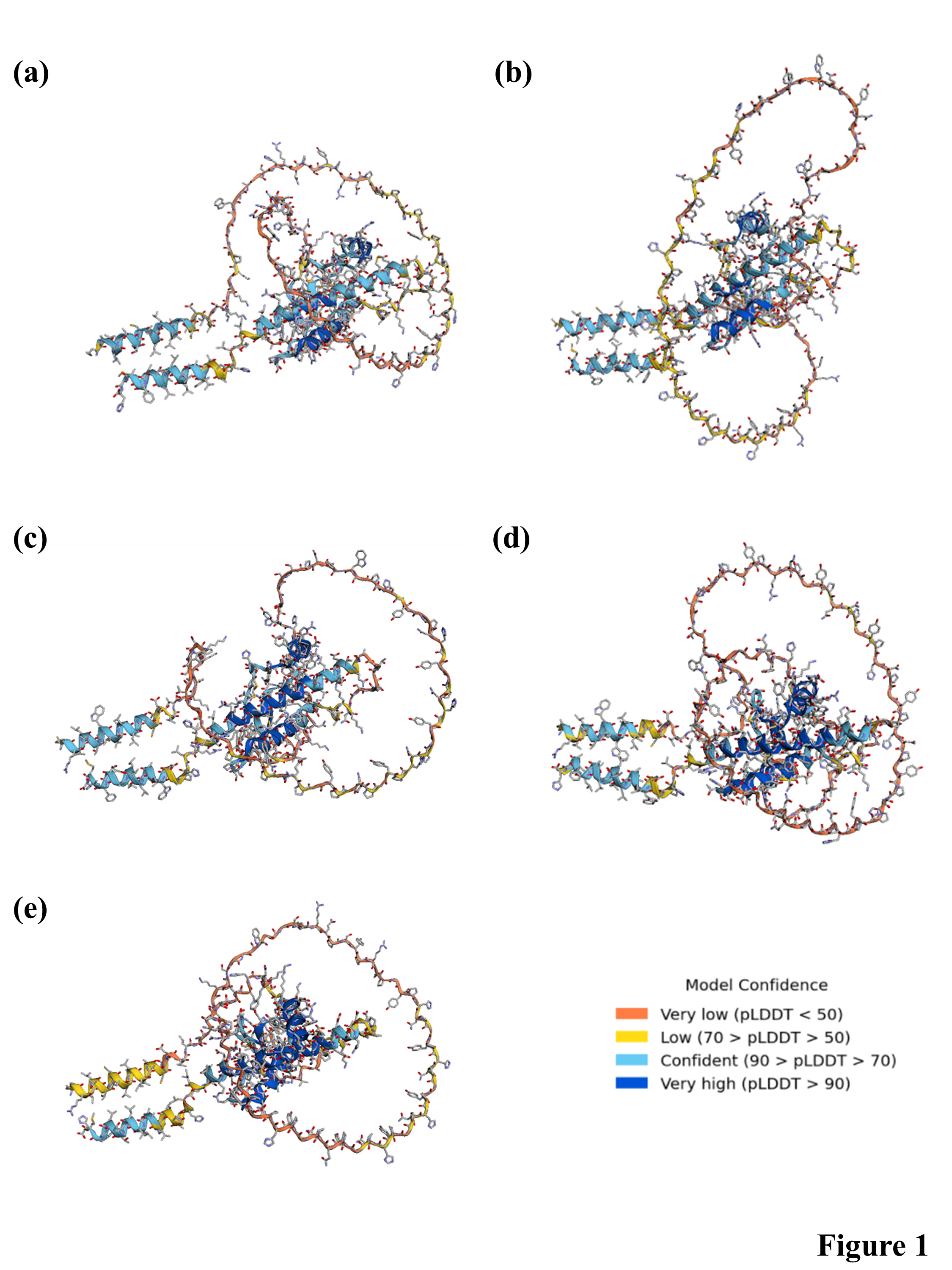

Supplement: Supplementary Figure 1 — (A) The tertiary structure of the chicken prion protein (PrP). (B) The tertiary structure of turkey PrP. (C) The tertiary structure of the goose PrP. (D) The tertiary structure of duck PrP. (E) The tertiary structure of the quail PrP. pLDDT: predicted local distance difference test. [file Image_1.TIF]
